# Fulminant Hepatic Failure Secondary to Primary Hepatic Angiosarcoma

**DOI:** 10.1155/2015/869746

**Published:** 2015-02-28

**Authors:** Ayokunle T. Abegunde, Efe Aisien, Benjamin Mba, Rohini Chennuri, Marin Sekosan

**Affiliations:** ^1^Department of Medicine, John H. Stroger Jr. Hospital of Cook County, Chicago, IL 60612, USA; ^2^Department of Medicine, Section of Digestive Diseases and Nutrition, Oklahoma University Health Sciences Center, Oklahoma City, OK 73104, USA; ^3^Department of Pathology, John H. Stroger Jr. Hospital of Cook County, Chicago, IL 60612, USA

## Abstract

*Background*. Hepatic angiosarcoma is a rare and aggressive tumor that often presents at an advanced stage with nonspecific symptoms. *Objective*. To report a case of primary hepatic angiosarcoma in an otherwise healthy man with normal liver function tests two months prior to presenting with a short period of jaundice that progressed to fulminant hepatic failure. *Methods*. Case report and review of literature. *Conclusion*. This case illustrates the rapidity of progression to death after the onset of symptoms in a patient with hepatic angiosarcoma. Research on early diagnostic strategies and newer therapies are needed to improve prognosis in this rare and poorly understood malignancy with limited treatment options.

## 1. Case Report

**Figure 1:**
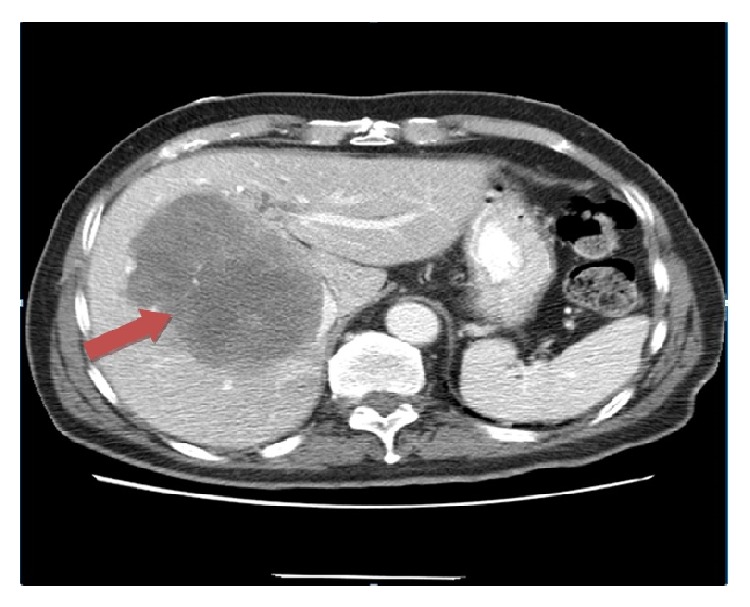
Computerized abdominal tomography scan showing the 13 × 11 × 10 cm hepatic mass with small dilated bile ducts surrounding the mass.

**Figure 2:**
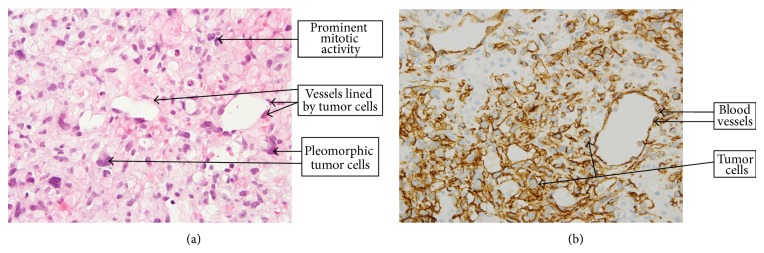
(a) Angiosarcoma: H&E section (600x magnification). (b) CD34 immunostain highlights tumor cells along with blood vessels (400x magnification).

A 75-year-old man presented to our hospital with three-week history of anorexia, abdominal fullness, chills, and three-day history of jaundice. He was diagnosed with AV nodal reentry tachycardia (AVNRT) six months earlier which terminated with the Valsalva maneuver. His medication included Diltiazem and Aspirin. He had no further episodes of AVNRT and liver enzymes were normal at routine follow-up in the cardiology clinic two months before presentation. On admission he was afebrile and reported no abdominal pain, hematemesis, melena, or pale stools. Physical examination revealed a frail looking man with skin and scleral icterus, normal heart sounds, and blood pressure. Abdominal examination revealed epigastric tenderness without organomegaly or ascites. Laboratory tests were significant for total bilirubin 9 mg/dL, albumin 4.0 g/dL, alkaline phosphatase 383 U/L, gamma-glutamyl transferase 701 U/L, aspartate aminotransferase, 104 U/L, alanine aminotransferase 206 U/L, and lactate dehydrogenase 451 U/L. Carcinoembryonic antigen (CEA) and alpha fetoprotein (AFP) levels were normal and CA19-9 was 38.8 IU/mL. Human immunodeficiency virus (HIV) and hepatitis panel were negative. Chest X-ray was normal and ECG showed sinus tachycardia. Abdominal ultrasound scan was negative for cholelithiasis and cholecystitis. Contrast enhanced triple-phase computerized tomography scan of his abdomen showed a hepatic mass measuring 13 × 11 × 10 cm with small dilated bile ducts surrounding the mass (Figure 1). There was no common bile duct (CBD) dilatation. Liver abscess, metastases, atypical hepatocellular carcinoma (HCC), and hepatic cystadenocarcinoma were considered in the differential diagnosis. He developed a fever on the second day of hospitalization and was started on piperacillin-tazobactam. Pan cultures were negative and he continued to spike fevers despite intravenous antibiotics. Eventually, his fever subsided and he underwent CT guided percutaneous biopsy of the hepatic mass. He was discharged home uneventfully 4 days later. Histopathology confirmed high grade primary angiosarcoma of the liver (Figures 2(a) and 2(b)). He was seen in clinic 2 weeks later at his posthospital follow-up appointment to discuss the biopsy result and management options. Unfortunately, he was admitted to another hospital within 1 month of discharge and died of fulminant hepatic failure.

## 2. Discussion

### 2.1. Epidemiology

Hepatic angiosarcoma (HAS) is a very rare and aggressive tumor that accounts for about 1.8–2% of all primary liver cancers. Epidemiologically, it is associated with exposure to polyvinyl chloride (PVC), thorium dioxide (thorothrast), arsenic, and inorganic copper [1, 2]. Hepatic angiosarcoma has also been associated with anabolic steroids, hemochromatosis, and neurofibromatosis (NF-1) [3, 4]. Based on studies from national registries of the United States and United Kingdom, the time period from risk factor exposure to development of disease is about 25–35 years, with peak incidence in the 6th and 7th decade of life [1, 2]. Hepatic angiosarcoma affects more males than females (3 : 1) and most cases are associated with occupational exposure or iatrogenic exposure to thorothrast during radiological investigations [1, 2]. There appears to be a younger age of onset and higher incidence of hepatic angiosarcoma in Asia, particularly based on reports from China, Korea, and Japan [5–7]. However, these reports are mainly hospital based and should be interpreted with caution. Median survival is about 6 months with treatment and at best 16 months [5]. There are recent reports of longer survival following hepatic resection of single tumors detected at a relatively early stage when hepatic resection is feasible [8]. We report an illustrative case of primary angiosarcoma of the liver in an otherwise healthy man who presented with a short onset of jaundice that progressed to fulminant hepatic failure. Our patient had none of the risk factors associated with hepatic angiosarcoma and his liver enzymes were normal two months prior to presentation providing no clue to suspect underlying liver pathology (Table 1).

### 2.2. Clinical Presentation and Diagnosis

The presenting symptoms of hepatic angiosarcoma are nonspecific and may be confused with any other hepatobiliary disorder [9]. The most commonly reported symptoms include abdominal pain, malaise, jaundice, ascites, and massive hepatomegaly [9]. In view of the long latency between exposure and development of the tumor the full extent of occurrence and symptoms may not be known for about thirty-five years [1]. Progression of disease is often fast and fatal with death occurring within a few weeks from intra-abdominal bleeding, hepatic coma, fulminant hepatic failure, or sepsis [9, 10]. Diagnosis is challenging because most patients such as the patient described in this report do not have any identifiable risk factors [9, 10]. Second, radiological investigations may reveal an inconclusive multifocal or focal hepatic mass that has broad differential diagnoses such as liver abscess, hepatocellular cancer (HCC), metastases, primary hepatic lymphoma, hepatic cystadenocarcinoma, hemangioblastoma and hepatic epithelioid hemangioendothelioma (HEHE) (Table 2). Third, patients present at an advanced stage with such severe illness that diagnoses is made at autopsy. Necropsy studies have showed considerable, often massive, replacement of liver tissue by unifocal or multifocal tumor with necrosis and hemorrhage [1]. In patients with reasonable performance status, liver biopsy is often required to make the diagnosis. There are two main approaches to liver biopsy; percutaneous and transjugular. There is some controversy regarding what the best approach is. Ultrasound guided percutaneous biopsy has been associated with a few reports of fatal intra-abdominal bleeding [11, 12]. The transjugular approach has been reported to be safer and associated with less complications from intra-abdominal bleeding. However, percutaneous biopsy has been shown to be faster, safe, and effective in establishing the diagnosis of hepatic angiosarcoma [13]. The classic pathologic findings on microscopic analysis are proliferation of single or multilayered tumor cells along liver sinusoids, atrophy of liver cell cords, and a solid tumor nest composed of spindle shaped and polyhedral cells without significant vascular spaces. Immunohistochemical staining with factor VIII-related antigen, CD31, and CD34 provides additional evidence in support of the diagnosis [9, 10]. The differentials in the histopathologic diagnosis of hepatic angiosarcoma include epithelioid hemangioendothelioma, hepatocellular carcinoma (hemorrhagic), Kaposi sarcoma (in HIV-positive cases), undifferentiated sarcoma (most common in children), and peliosis hepatis in cases of anabolic steroid use and bacillary angiomatosis, among other causes. The macroscopic (gross), microscopic (histological), and immunohistochemical features of these entities in the differential are discussed in Table 3 [14, 15].

### 2.3. Hepatic Angiosarcoma and Liver Failure

Fulminant liver failure (FHF) due to HAS is very rare and the mechanism of liver failure is multifactorial. There have been six cases of FHF secondary to HAS reported in the literature to date and the available evidence suggests that a combination of hepatic ischemia leading to parenchymal infarction, vascular occlusion of portal vein flow by tumor, thrombi, and nonocclusive infarction of the liver due to shock from secondary causes such as sepsis or cardiac dysfunction may play a role [16–19]. Disseminated intravascular coagulation (DIC) is rare in HAS, occurring in less than 5% of cases [20, 21]. A rare association with Kasabach-Merritt syndrome was reported in one case [22]. Mortality in the reported cases was secondary to consumptive coagulopathy and massive intra-abdominal bleeding [20–22]. Other mechanisms of FHF secondary primary or metastatic liver cancer include (1) localized submassive liver cell necrosis resulting from sudden reduction in portal perfusion which has been inadequate because of tumor thrombosis, combined with hypotension of hepatic arteries and (2) malignant infiltration and replacement of hepatocytes by tumor leading to secondary necrosis of hepatocytes and development of liver failure [23–25]. In our opinion, malignant infiltration and secondary necrosis was the most likely mechanism leading to the development of liver failure in our patient.

### 2.4. Management

Treatment of the tumor to date is empirical [26]. Radical surgery with adjuvant chemotherapy appears to be the most promising treatment with the possibility of cure if the tumor is solitary and is diagnosed early with reported median survival of about 17 months [5, 26, 27]. There are few case reports describing extended survival beyond one year following surgical resection [8, 17, 28]. However, no standardized surgical procedure has been established, and it should be noted that the average age of patients operated in the only major series was 55.5 years [8]. The current management approach to primary liver cancer is the determination of eligibility for liver transplant. Liver transplantation (LT) has showed survival benefit in the management of other primary liver cancers such as HCC and HEHE [29]. However, LT has not shown a survival benefit in HAS when compared with surgical resection in reported studies [8, 19, 28, 30, 31]. The dismal survival reported after liver transplantation for HAS (mean 7.2 ± 2.6 months) with no patient surviving after 23 months is attributed to early recurrence after transplantation (mean 5.0 ± 2.6 months) and poor performance status of patients with HAS [31]. Transarterial chemoembolization (TACE) has been used as palliative therapy to control intra-abdominal bleeding in patients with hepatic angiosarcoma [32]. Two small case series comprised of a total of 9 patients with HAS treated with different chemotherapy regimens have shown limited efficacy in treating hepatic angiosarcoma; survival from time of diagnosis ranged from <3 months to >53 months [10, 33, 34]. Targeted therapies such as bevacizumab and sorafenib have been shown to have limited efficacy in treating primary angiosarcoma of other organs; however only one case of HAS was included in these studies [35, 36]. There are currently very limited studies investigating new therapies or early diagnostic strategies [26]. However, prevention of occupational or iatrogenic exposure to established risk factors such as PVC is effective in reducing the incidence of HAS [37, 38]. This was demonstrated following an outbreak of four cases of HAS at a PVC factory in the United States between 1967 and 1973; an industry-wide law to reduce average exposure to vinyl chloride was key to reducing exposure to PVC and new cases of HAS among PVC factory workers [38]. Such policy decisions with appropriate enforcement by law are a potential strategy for reducing exposure to established risk factors in countries without such legislation and rapid industrialization. Research on early diagnostic strategies and newer therapies are needed to improve prognosis. However, the rarity of this disease inhibits progress.

## Figures and Tables

**Table 1:** Laboratory tests two months prior to presentation, at presentation, and at the time of patient's death.

Laboratory tests	Normal range	2 months prior to presentation	At presentation	Time of death
Liver function tests				
Total bilirubin	0.3–1.2 mg/dL	0.9	9.8	27
Direct bilirubin	0–0.3 mg/dL	0.2	6.3	18.5
Alkaline phosphatase	36–92 U/L	81	383	1120
Gamma-glutamyl transferase	0–30 U/L	58	701	1200
Aspartate aminotransferase	0–35 U/L	25	104	5522
Alanine aminotransferase	0–35 U/L	21	206	2313
Prothrombin time	11–13 sec	14.1	14.8	43.2
Partial thromboplastin time	25–35 sec	27.5	34.6	69.9
International normalized ratio		1.10	1.16	4.97
Lactate dehydrogenase (LDH)	60–100 U/L	190	451	20000
Peripheral blood cell count				
Hemoglobin, g/L	12.6–16.8	14.4	14.1	5.5
White blood cell count, /L	3.7–10.5 × 10^9^	5.4	6.5	26
Platelet count, /L	167–400 × 10^9^	144	143	22
Blood chemistry				
Sodium, mmol/L	135–144	145	139	123
Potassium, mmol/L	3.5–5	4.6	5.1	5.4
Chloride, mmol/L	100–110	110	104	95
Bicarbonate, mmol/L	23–31	24	24	10
Blood urea nitrogen, mmol/L (mg/dL)	2.85–7.14 (7.98–20)	15	20	80
Creatinine, mmol/L (mg/dL)	53.0–123.7 (0.6–1.4)	0.8	1.2	6.3

**Table 2:** Differential diagnosis of a focal hepatic mass.

Diagnosis	Clinical features	Radiographic appearance
Hemangioma	Most often asymptomatic, but can present with right upper quadrant pain and fullness. Hepatomegaly, hepatic bruit, jaundice, GI bleed, and fever may occur.	*Ultrasound*: Well demarcated homogenous, hyperechoic mass. *CT scan*: (triple phase with delayed imaging) (i) Hypodense on noncontrast CT. (ii) Peripheral enhancement on arterial phase. (iii) Centripetal contrast enhancement on venous and delayed phases.

Focal nodular hyperplasia	Largely benign symptomatology. Abdominal discomfort and palpable liver mass sometimes seen.	*Ultrasound*: Variably hyper-, hypo-, or isoechoic. A central scar is identified in 20% of cases. *CT scan*: Noncontrast: Hypo- or isodense (central scar in 1/3 of patients.) Contrast study: Hyperdense during arterial phase and isodense during the portal venous phase. *MRI*: T1: Isointense T2: Isointense to slightly hyperintense with generally hyperintense central scar. Gadolinium: Hyperintense. *Nuclear imaging*: Technetium sulfur colloid scanning due to presence of Kupffer cells; 80% of lesions show active uptake.

Hepatic cystadenoma	RUQ discomfort and pain; anorexia. Frequently asymptomatic and an incidental finding.	*Ultrasound*: Anechoic lesions with internal septations. *CT*: Hypodense lesions with focal enhancement postcontrast. *MRI*: Hypointense on T1-weighted images. Hyperintense on T2-weighted images.

Hepatic abscess	Fever, abdominal pain, nausea, vomiting, and anorexia.	*Ultrasound*: Hypoechoic masses with irregularly shaped borders. *CT scan*: Hypodense masses with peripheral enhancement post-contrast. (Enhanced ring sign.)

Hepatic adenoma	Episodic abdominal pain. Frequently an incidental finding. An abdominal mass is felt 30% of the time, hepatomegaly (25%). Rarely, jaundice.	*Ultrasound*: Often nonspecific. It could have well demarcated hyper echoic appearance or heterogeneous due to intratumoral bleeding. *CT scan*: *Noncontrast*: Well-defined hypo- or isodense lesions. *Contrast*: Arterial phase, peripheral enhancement. Portal venous phase: Centripetal flow. Late phase: Isodense initially, then hypodense. *MRI*: Well-defined, but highly variable appearance. Most are hyperintense on T1-weighted and T2 images. There is early enhancement with gadolinium, but the lesion. *Nuclear imaging*: Most adenomas (>75%) do not take up Technetium, and it more frequently appears as a cold spot.

Hepatic cystadenocarcinoma	Jaundice, abdominal pain, weight loss, and ascites due to portal vein compression.	*Ultrasound*: Anechoic mass with echogenic internal septations. *CT scan*: Multiloculated hypodense masses with possible coarse calcifications. *MRI*: Fluid-containing multilocular cyst with homogenous low signal intensity on T1 and homogenous high signal intensity on T2. *Nuclear scans*: No role.

Hepatocellular carcinoma	RUQ pain, weight loss, decompensated liver disease; paraneoplastic phenomena such as erythrocytosis, hypercalcemia, and watery diarrhea.	*Ultrasound*: Poorly defined margins and coarse, irregular internal echoes. Small tumors tend to be hypoechoic but become isoechoic or hyperechoic with increasing size. *CT scan*: Hypervascular lesion with arterial enhancement and rapid washout during the portal venous phase. *MRI*: HCC appears as high intensity lesions on T2 and low intensity on T1-weighted images. MRI is better than CT and USS in cirrhotic patients in differentiating regenerative nodules from HCC.

Metastases	Abdominal pain, RUQ tenderness, jaundice, fever, weight loss, anorexia, hepatomegaly, and ascites.	*Ultrasound*: Generally nonspecific appearance of liver mets. Usually, multiple hepatic nodules of myriad sizes which could be isoechoic, hyperechoic, or hypoechoic. *CT scan*: Hepatic metastases appear in a plurality of ways. The majority are hypoattenuating (due to hypervascularity). Noncontrast images are useful in detecting calcification and hemorrhage. *MRI*: Metastatic lesions are hyperintense on T1-weighted images and hypointense on T2-weighted. The morphologic characteristics on T2 that suggest metastases are as follows: Heterogeneous signal intensity with irregular and indistinct outer margins. (i) Smooth or irregular central area of high signal intensity with surrounding ring of lower intensity.

Cholangiocarcinoma	Painless jaundice, pruritus, abdominal pain, weight loss, fever, clay-colored stools, and dark urine.	*Ultrasound*: Biliary dilatation and large hilar lesions. Smaller lesions are more challenging to visualize. Patients with primary sclerosing cholangitis may not show any ductal dilatation due to ductal fibrosis. *CT scan*: Ductal dilatation with possible mass lesions and lymphadenopathy. *MRI/MR cholangiography*: MRI shows liver parenchyma better than CT, and it also offers better imaging of bile ducts by cholangiography. With angiography, it is an excellent tool for staging. Lesions appear hypointense on T1- and hyperintense on T2-weighted imaging.

Hemangioendothelioma	Frequently asymptomatic. RUQ pain, weight loss, and hepatomegaly.	*Ultrasound*: Predominantly hypoechoic, but could be mixed and even hyperechoic. *CT scan*: Multiple hypodense lesions, frequently peripheral. Often, smaller lesions are seen to coalesce to form larger ones. *MRI*: T1-weighted: Hypointense lesions. T2-weighted: Hyperintense lesions. Postgadolinium: Occasional thin peripheral hypointense rim.

Primary hepatic lymphoma	Hepatomegaly, splenomegaly; fever, night sweats, weight loss, and lymphadenopathy.	*Ultrasound*: Usually hypoechoic lesions. *CT scan*: Single, well-defined hypodense lesion. Nonspecific postcontrast appearance. *MRI*: T1-weighted: Hypointense. T2-weighted: Variable intensity. Gadolinium: It may show faint rim enhancement.

Hemangioblastoma	Extremely rare liver lesion. Seen in the setting of von Hippel-Lindau syndrome.	*Ultrasound*: Usually, multiple hyperechoic foci, but this is variable. *CT scan*: Variable appearances. Noncontrast: Generally appearing as hypodensities. Postcontrast: Early peripheral enhancement and partial centripetal isodense filling.

(1) Schwartz and Kruskal [39].

**Table 3:** Histopathologic features in the differential diagnosis of hepatic angiosarcoma.

Tumor	Gross characteristics	Microscopy/histologic features	Immunohistochemistry
Angiosarcoma	Ill-defined, diffusely infiltrative spongy nodules with hemorrhage. Extensive and diffuse involvement of liver.	The tumor consists of malignant endothelial cells which grow along preexisting vascular channels and hepatic sinusoids. It shows solid and pseudopapillary patterns. Necrosis and hemorrhage present. Plump spindle cells with large pleomorphic nuclei.	Positive for CD31, CD34, factor VIII Weibel-Palade bodies on electron microscopy.

Epithelioid hemangioendothelioma	Multiple, tan-gray, firm, circumscribed and focally confluent nodules up to 12 cm with infiltrative borders. It may show central calcification or ossification.	Tumor exhibits zonal pattern, with central sclerosis or hyalinization and tumor cells at the periphery in a sinusoidal proliferation. Tumor forms papillary tufting and glomeruloid structures within portal vein branches. Eosinophilic epithelioid tumor cells typically show vacuolated signet-ring-like features representing intracytoplasmic lumina sometimes containing erythrocytes.	Positive for factor VIII, CD31, CD34, cytokeratin (50%) Weibel-Palade bodies, and intermediate filaments on electron microscopy.

Hepatocellular carcinoma (HCC)	Solitary, multinodular or diffusely infiltrative soft, yellow-green or reddish mass in a background of cirrhosis. Smaller satellite nodules around large mass. High propensity of tumor to invade into the portal veins. Hemorrhage and necrosis are common.	Major histologic patterns are trabecular (plate-like), pseudoglandular (acinar) and compact (solid) types. Cells are polygonal with distinct cell membranes, abundant granular eosinophilic cytoplasm, higher nucleocytoplasmic ratio than normal, round nuclei with coarse chromatin and may have prominent nucleoli. Presence of sinusoidal vessels surrounding tumor cells is an important diagnostic feature. Intranuclear inclusions including eosinophilic hyaline bodies, Mallory hyaline, and fat droplets may be present.	Positive for HepPar1 (90% of all HCCs) and glypican-3, canalicular pattern of staining with polyclonal CEA, AFP (25%).

Kaposi sarcoma (aggressive variant associated with AIDS)	Hemorrhagic multifocal spongy nodules 5–7 cm.	Lesions centered on portal tracts with poorly vasoformative spindle-cell proliferation accompanied by red blood cell extravasation and focal deposition of hemosiderin. Cytoplasmic eosinophilic hyaline globules are a typical finding.	Positive for membranous/cytoplasmic CD31 and CD34 and nuclear HHV8.

Undifferentiated sarcoma (most common in children age 6–10 years)	Well-demarcated, solitary, unencapsulated lesion. Cut surface is variegated with solid and cystic/gelatinous areas, with necrosis and hemorrhage.	Tumor consists of loosely arranged, spindle-pleomorphic cells embedded in an abundant mucopolysaccharide-rich myxoid matrix. Dilated bile ducts and PAS-positive diastase-resistant globules found within the tumor cells; tumor not particularly vascular.	Positive for Vimentin, focally positive for Keratin; CD31 negative.

Peliosis hepatis (associated with exposure to anabolic steroids, tuberculosis, and AIDS)	Honeycombed liver with multiple round, red-purple, blood filled spaces, range from 1mm to several cm.	Lesion consists of blood-filled spaces surrounded by a pseudocapsule of fibrin and early collagen. Rarely endothelial lining visible. *B. henselae* (bacillary angiomatosis) cases have small blood vessel proliferation and spindle cells.	Positive for Warthin-Starry stain (*Bartonella henselae* infection in HIV patients).

(1) Gattuso et al. [14].

(2) Rosai [15].

## References

[1] Lee F. I., Smith P. M., Bennett B., Williams D. M. J. (1996). Occupationally related angiosarcoma of the liver in the United Kingdom 1972–1994. *Gut*.

[2] Falk H., Herbert J., Crowley S. (1981). Epidemiology of hepatic angiosarcoma in the United States: 1964–1974. *Environmental Health Perspectives*.

[3] Lederman S. M., Martin E. C., Laffey K. T., Lefkowitch J. H. (1987). Hepatic neurofibromatosis, malignant schwannoma, and angiosarcoma in von Recklinghausen's disease. *Gastroenterology*.

[4] Falk H., Thomas L. B., Popper H., Ishak K. G. (1979). Hepatic angiosarcoma associated with androgenic-anabolic steroids. *The Lancet*.

[5] Arima-Iwasa S., Chijiiwa K., Makino I., Tanabe R., Ohuchida J., Kondo K. (2007). A case of hepatic angiosarcoma surviving for more than 16 months after hepatic resection. *Hepato-Gastroenterology*.

[6] Ho S. Y., Tsai C. C., Tsai Y. C., Guo H. R. (2004). Hepatic angiosarcoma presenting as hepatic rupture in a patient with long-term ingestion of arsenic. *Journal of the Formosan Medical Association*.

[7] Huang N. C., Wann S. R., Chang H. T., Lin S. L., Wang J. S., Guo H. R. (2011). Arsenic, vinyl chloride, viral hepatitis, and hepatic angiosarcoma: a hospital-based study and review of literature in Taiwan. *BMC Gastroenterology*.

[8] Duan X. F., Li Q. (2012). Primary hepatic angiosarcoma: a retrospective analysis of 6 cases. *Journal of Digestive Diseases*.

[9] Molina E., Hernandez A. (2003). Clinical manifestations of primary hepatic angiosarcoma. *Digestive Diseases and Sciences*.

[10] Kim H. R., Rha S. Y., Cheon S. H., Roh J. K., Park Y. N., Yoo N. C. (2009). Clinical features and treatment outcomes of advanced stage primary hepatic angiosarcoma. *Annals of Oncology*.

[11] Drinković I., Brkljačić B. (1996). Two cases of lethal complications following ultrasound-guided percutaneous fine-needle biopsy of the liver. *CardioVascular and Interventional Radiology*.

[12] Hertzanu Y., Peiser J., Zirkin H. (1990). Massive bleeding after fine needle aspiration of liver angiosarcoma. *Gastrointestinal Radiology*.

[13] Wong J. W., Bedard Y. C. (1992). Fine-needle aspiration biopsy of hepatic angiosarcoma: report of a case with immunocytochemical findings. *Diagnostic Cytopathology*.

[14] Gattuso P., Reddy V. B., David O., Spitz D. J., Haber M. H., Yeh M. W., Swanson P. E. (2010). Hepatobiliary system. *Differential Diagnosis in Surgical Pathology*.

[15] Rosai J. (2011). Liver—tumors and tumorlike conditions. *Rosai and Ackerman's Surgical Pathology*.

[16] Montell García M., Romero Cabello R., Romero Feregrino R. (2012). Angiosarcoma of the liver as a cause of fulminant liver failure. *BMJ Case Reports*.

[17] Egea Valenzuela J., López Poveda M. J., Pérez Fuenzalida F. J., Garre Sánchez C., Martínez Barba E., Carballo Álvarez F. (2009). Hepatic angiosarcoma. Presentation of two cases. *Revista Espanola de Enfermedades Digestivas*.

[18] Bhati C. S., Bhatt A. N., Starkey G., Hubscher S. G., Bramhall S. R. (2008). Acute liver failure due to primary angiosarcoma: a case report and review of literature. *World Journal of Surgical Oncology*.

[19] Akdoğan M., Gürakar A., Sharago S. (2002). Unusual presentation of hepatic vascular tumors as fulminant hepatic failure. *Turkish Journal of Gastroenterology*.

[20] Locker G. Y., Doroshow J. H., Zwelling L. A., Chabner B. A. (1979). The clinical features of hepatic angiosarcoma: a report of four cases and a review of the English literature. *Medicine*.

[21] Lespi P. J., Alvarez G. R., Iannariello M. B., Wisnowski C. (1997). Hepatic angiosarcoma with disseminated intravascular coagulation. *Medicina*.

[22] Alliot C., Tribout B., Barrios M., Gontier M. F. (2001). Angiosarcoma variant of Kasabach-Merritt syndrome. *European Journal of Gastroenterology and Hepatology*.

[23] Okuda K., Musha H., Kanno H. (1976). Localized submassive liver cell necrosis as a terminal event of liver carcinoma. *Cancer*.

[24] Rajvanshi P., Kowdley K. V., Hirota W. K., Meyers J. B., Keeffe E. B. (2005). Fulminant hepatic failure secondary to neoplastic infiltration of the liver. *Journal of Clinical Gastroenterology*.

[25] Alexopoulou A., Koskinas J., Deutsch M., Delladetsima J., Kountouras D., Dourakis S. P. (2006). Acute liver failure as the initial manifestation of hepatic infiltration by a solid tumor: report of 5 cases and review of the literature. *Tumori*.

[26] Zheng Y. W., Zhang X. W., Zhang J. L. (2014). Primary hepatic angiosarcoma and potential treatment options. *Journal of Gastroenterology and Hepatology*.

[27] Zhou Y. M., Li B., Yin Z. F. (2010). Results of hepatic resection for primary hepatic angiosarcoma in adults. *Medical Science Monitor*.

[28] Matthaei H., Krieg A., Schmelzle M. (2009). Long-term survival after surgery for primary hepatic sarcoma in adults. *Archives of Surgery*.

[29] Eghtesad B., Aucejo F. (2014). Liver transplantation for malignancies. *Journal of Gastrointestinal Cancer*.

[30] Timaran C. H., Grandas O. H., Bell J. L. (2000). Hepatic angiosarcoma: long-term survival after complete surgical removal. *The American Surgeon*.

[31] Orlando G., Adam R., Mirza D. (2013). Hepatic hemangiosarcoma: an absolute contraindication to liver transplantation–the European liver transplant registry experience. *Transplantation*.

[32] Park Y. S., Kim J. H., Kim K. W. (2009). Primary hepatic angiosarcoma: imaging findings and palliative treatment with transcatheter arterial chemoembolization or embolization. *Clinical Radiology*.

[33] Dannaher C. L., Tamburro C. H., Yam L. T. (1981). Chemotherapy of vinyl chloride-associated hepatic angiosarcoma. *Cancer*.

[34] Stacchiotti S., Palassini E., Sanfilippo R. (2012). Gemcitabine in advanced angiosarcoma: a retrospective case series analysis from the Italian rare cancer network. *Annals of Oncology*.

[35] Agulnik M., Yarber J. L., Okuno S. H. (2013). An open-label, multicenter, phase II study of bevacizumab for the treatment of angiosarcoma and epithelioid hemangioendotheliomas. *Annals of Oncology*.

[36] Ray-Coquard I., Italiano A., Bompas E. (2012). Sorafenib for patients with advanced angiosarcoma: aA phase II trial from the french sarcoma group (GSF/GETO). *Oncologist*.

[37] Office of Technology Assessment (1995). *Gauging Control Technology and Its Regulatory Impacts in Occupational Safety and Health*.

[38] Falk H., Baxter P. J., Peto R., Schneiderman M. (1981). Hepatic angiosarcoma registries: implications for rare tumor studies. *Banbury Report no. 9: Quantification of Occupational Cancer*.

[39] Schwartz J. M., Kruskal J. B. http://www.uptodate.com/contents/solid-liver-lesions-differential-diagnosis-and-evaluation.

